# Platelet trajectory patterns and 28-day mortality in ICU stroke patients: a retrospective cohort study based on the MIMIC-IV database

**DOI:** 10.1186/s12883-026-05222-8

**Published:** 2026-08-06

**Authors:** Tao Chen, Rui Liang, Riping Xu, Can Chen

**Affiliations:** 1 Jinan University, Guangzhou, 510632 China; 2 Department of Cardiology, Affiliated Hospital of Guangdong Medical University, Zhanjiang, 524000 Guangdong China; 3 Department of Neurology, Institute of Neuroscience, Key Laboratory of Neurogenetics and Channelopathies of Guangdong Province and the Ministry of Education of China, The Second Affiliated Hospital of Guangzhou Medical University, Guangzhou, 510260 China; 4 Department of Anesthesiology, Affiliated Hospital of Guangdong Medical University, Zhanjiang, 524000 Guangdong China

**Keywords:** Platelet, Stroke, ICU, Trajectory, Mortality, MIMIC-IV

## Abstract

**Background:**

Stroke remains a leading cause of death and disability worldwide. Platelet abnormalities are common in critically ill patients, but dynamic trajectory patterns and their prognostic value in intensive care unit (ICU) stroke patients remain unclear.

**Methods:**

This retrospective cohort study included 1,562 ICU stroke patients from the Medical Information Mart for Intensive Care (MIMIC-IV) database (version 3.1). Latent class trajectory models (LCTM) were used to identify platelet trajectory patterns during the first 5 days of ICU admission. Cox proportional hazards regression was performed to assess the association between trajectory groups and 28-day mortality. Exploratory effect decomposition analysis was conducted to explore the relative contribution of baseline platelet levels and subsequent platelet decline to prognosis.

**Results:**

Four distinct platelet trajectory groups were identified: High-Declining (14.7%), Moderate-Low Stable (52.4%), Low-Rising (4.5%), and Moderate-High Stable (28.4%). The High-Declining group had the highest 28-day mortality (34.1% vs. 20.4% in the Moderate-Low Stable group, *P* < 0.001). After multivariable adjustment, the High-Declining group was associated with a 56% increase in mortality risk compared with the Moderate-Low Stable group (hazard ratio [HR] = 1.56, 95% confidence interval [CI]: 1.17–2.07, *P* = 0.002). Exploratory effect decomposition analysis revealed a suppression pattern: the direct protective effect of higher Day 2 platelet was offset by an indirect harmful effect mediated through greater subsequent platelet decline (Average Component Mediation Effect [ACME] = 0.017 on the probability-difference scale, 95% CI: 0.010–0.026, *P* < 0.001), suggesting that rapid platelet decline may reflect a high-risk phenotypic pattern associated with this trajectory group.

**Conclusions:**

Platelet trajectory patterns are associated with 28-day mortality and may serve as prognostic indicators in ICU stroke patients. A high baseline platelet level with rapid decline represents the highest-risk phenotype and, if confirmed in future prospective studies, may help identify patients requiring closer clinical evaluation to screen for underlying deteriorating conditions.

**Supplementary Information:**

The online version contains supplementary material available at https://doi.org/10.1186/s12883-026-05222-8.

## Introduction

Stroke is the second leading cause of death worldwide and a major cause of long-term disability [1–3]. According to the Global Burden of Disease Study, there were approximately 12.2 million new stroke cases and 101 million stroke survivors globally in 2019 [1]. Stroke patients admitted to the ICU are critically ill with high mortality rates; early identification of high-risk patients is crucial for improving prognosis.

Platelets play multiple roles in the pathophysiology of stroke [4–8]. In acute ischemic stroke, platelet aggregation and thrombosis are the core mechanisms leading to cerebrovascular occlusion; in hemorrhagic stroke, platelet dysfunction and coagulation abnormalities contribute to hematoma expansion. In addition, platelets are involved in inflammatory responses, endothelial function regulation, and tissue repair [4, 5]. Platelets regulate the inflammatory microenvironment by releasing various bioactive substances, thereby influencing secondary brain injury after stroke [6, 7].

Abnormal platelet counts are common in ICU patients. Studies have shown that approximately 20–50% of ICU patients develop thrombocytopenia (< 100 × 10⁹/L), while thrombocytosis (> 450 × 10⁹/L) occurs in about 5–15% of cases [9–16]. Thrombocytopenia is associated with increased bleeding risk, higher transfusion requirements, and elevated mortality; thrombocytosis is associated with increased thrombotic event risk [9, 10, 15, 16].

Traditional studies have focused on single platelet measurements or simple categorizations, neglecting the dynamic changes in platelet counts [9–14]. However, trends in platelet changes during the acute disease course may reflect different pathophysiological mechanisms: a sustained decline suggests increased platelet consumption or bone marrow suppression, while gradual recovery may indicate favorable treatment response.

Previous studies have identified associations between platelet counts and stroke outcomes [17–20]. A systematic review by Leonaviciute et al. showed that thrombocytopenia at admission was associated with poor prognosis in acute stroke patients [17]. A meta-analysis by Wang et al. found a U-shaped relationship between admission platelet count and mortality in ischemic stroke [18]. However, these studies primarily focused on baseline platelet levels and lacked systematic investigation of dynamic change patterns.

In recent years, trajectory analysis methods have gained increasing attention in critical care research [21–30]. This approach can identify patient subgroups with similar change patterns. The Latent Class Trajectory Model (LCTM) is a statistical method based on finite mixture models [23–28]. Sjoding et al. used trajectory analysis to identify distinct inflammatory response phenotypes in sepsis patients [21]. Yoon et al. found that creatinine trajectories were closely associated with mortality in patients with acute kidney injury [22].

This study employed LCTM to identify platelet trajectory patterns in ICU stroke patients and examined the association between different patterns and mortality. Furthermore, we conducted effect decomposition analysis to explore the relative contributions of baseline platelet levels and subsequent change trends to prognosis [31–36]. This analysis helps address the clinically relevant question of whether baseline platelet information alone is sufficient, or whether early platelet change provides additional prognostic information in patients with high baseline platelet counts.

## Methods

This study was reported in accordance with the Strengthening the Reporting of Observational Studies in Epidemiology (STROBE) statement [37].

### Data source

This study used the publicly available de-identified Medical Information Mart for Intensive Care (MIMIC-IV) database (version 3.1) [38, 39], accessed via PhysioNet. MIMIC-IV v3.1 is a single-center retrospective electronic health record database maintained by the Massachusetts Institute of Technology, containing 364,627 unique patients, 546,028 hospital admissions, and 94,458 unique ICU stays from 2008 to 2022 [38, 39]. Patient identifiers were removed in accordance with the Health Insurance Portability and Accountability Act (HIPAA) Safe Harbor provision. As the data are publicly available and de-identified, the requirement for individual patient consent was waived.

#### Ethics approval and consent to participate

The establishment of the MIMIC-IV database was approved by the Institutional Review Board (IRB) of Beth Israel Deaconess Medical Center (BIDMC), which granted a waiver of informed consent for research use of the dataset (IRB #2001P001699). This retrospective study used publicly available de-identified data compliant with the HIPAA Safe Harbor provision; therefore, the requirement for individual patient consent was waived. Data access was obtained through PhysioNet after credentialing approval on 23 October 2023 for user “docchent” (CITI User ID: 12754809), following completion of CITI Program training (Completion Record ID: 59147883, valid from October 18, 2023 to October 18, 2027) and signing of the data use agreement.

### Study population

The patient selection flowchart is shown in Fig. S2. From the 94,458 ICU stays recorded in MIMIC-IV v3.1, 2,514 ICU stays met the inclusion criteria (age ≥ 18 years, ICU stay ≥ 48 h, and a documented acute cerebrovascular disease diagnosis). We then excluded 28 patients who died within 48 h of ICU admission, 924 patients with fewer than two platelet measurements within the first 5 days, and 0 patients with > 20% missing key variables, leaving 1,562 patients in the final analytic cohort. Of these, 1,461 (93.5%) were classified as ischemic stroke and 101 (6.5%) as hemorrhagic stroke.

Inclusion criteria were: (1) age ≥ 18 years; (2) ICU stay ≥ 48 h; (3) a documented diagnosis of acute cerebrovascular disease (ischemic or hemorrhagic stroke) during the hospitalization, identified using International Classification of Diseases (ICD) diagnosis codes (ICD-9-CM 430–438 and ICD-10-CM I60–I69), with stroke recorded as either the primary or a secondary diagnosis; and (4) serial platelet measurements (at least two platelet counts within the first 5 days after ICU admission). Exclusion criteria were: (1) death within 48 h of ICU admission; and (2) > 20% missing key data.

Key variables for the missing-data rule were defined a priori. They included demographics (age, sex), disease severity scores (Sequential Organ Failure Assessment [SOFA] score, Acute Physiology Score III [APSIII], Simplified Acute Physiology Score II [SAPSII]), Charlson Comorbidity Index, laboratory parameters (creatinine, blood urea nitrogen [BUN], sodium, potassium, glucose, international normalized ratio [INR], hemoglobin, white blood cell count), and therapeutic interventions (mechanical ventilation, continuous renal replacement therapy [CRRT], vasopressor use). A 20% missing-data threshold was chosen because values missing in more than one-fifth of a key variable are generally considered to compromise the validity of complete-case or imputed analyses.

Stroke cases were identified using International Classification of Diseases (ICD) diagnosis codes recorded in MIMIC-IV. We used the following code ranges to capture acute cerebrovascular disease: ICD-9-CM 430–438 and ICD-10-CM I60–I69 [40, 41]. Within each hospitalization (hadm_id), the cerebrovascular disease code with the smallest sequence number (seq_num) was used to classify stroke subtype. Codes were categorized as ischemic stroke if the most prioritized cerebrovascular diagnosis was ICD-10 I63 (cerebral infarction), I64 (stroke, not specified as hemorrhage or infarction), or ICD-9 433.x1 (occlusion and stenosis of precerebral arteries with cerebral infarction) / 436 (acute but ill-defined cerebrovascular disease). Codes were categorized as hemorrhagic stroke if the most prioritized diagnosis was ICD-10 I60 (subarachnoid hemorrhage), I61 (intracerebral hemorrhage), or I62 (other nontraumatic intracranial hemorrhage).

Using this algorithm, 1,461 patients (93.5%) were classified as ischemic stroke and 101 patients (6.5%) as hemorrhagic stroke. Because the cohort was overwhelmingly composed of ischemic stroke patients, the present study should be interpreted as primarily investigating ischemic stroke, although the original inclusion criteria permitted both subtypes to reflect the full spectrum of ICU stroke admissions captured in MIMIC-IV. Stroke was the primary admission diagnosis (seq_num = 1) in 48.6% of patients; in the remaining 51.4%, stroke was recorded as a secondary diagnosis during the hospitalization. This pattern is consistent with MIMIC-IV’s ICU-centric structure, in which critically ill patients are often admitted for complications or concurrent conditions while stroke is documented as a concurrent or contributory diagnosis.

We did not routinely exclude patients with transfusion, liver disease, or hypersplenism because these treatments and comorbidities reflect real-world ICU clinical practice and their precise timing or severity were not consistently captured in the MIMIC-IV extracts used for this analysis.

### Variable definitions and measurements

Primary exposure variable: platelet trajectory pattern, identified by LCTM based on the sequence of platelet counts during the first 5 days after ICU admission (Days 1–5).

Primary outcome variable: all-cause 28-day mortality. All-cause 28-day mortality was defined as death from any cause within 28 days of ICU admission. Death was identified from the MIMIC-IV admissions.deathtime (in-hospital death) and patients.dod (date of death, capturing post-discharge death). Patients alive at 28 days were censored at 28 days.

Covariates included: age and sex; disease severity scores (SOFA score [42], APSIII score, SAPSII score); baseline laboratory parameters (creatinine, BUN, sodium, potassium, glucose, INR, hemoglobin, white blood cell count); Charlson Comorbidity Index; and therapeutic interventions (mechanical ventilation, CRRT, vasopressor use). Race was not included in the final Cox model. The MIMIC-IV cohort is predominantly White, and the dichotomized White/non-White grouping would yield a very small non-White subgroup, leading to unstable effect estimates and limited confounding adjustment value beyond the included severity and comorbidity scores.

Treatment variables: Antiplatelet use and anticoagulant use during the hospitalization were identified from the MIMIC-IV prescriptions table. Antiplatelet agents included aspirin, clopidogrel, ticagrelor, prasugrel, and dipyridamole. Anticoagulants included heparin, warfarin, apixaban, rivaroxaban, dabigatran, and edoxaban. These variables were treated as binary exposure indicators (any prescription vs. none during the index hospitalization) and were summarized in the baseline characteristics table; they were not added as covariates in the primary Cox models because of the high prevalence of anticoagulant use (98.0%), the strong correlation between antiplatelet/anticoagulant therapy and stroke subtype, and the risk of over-adjustment for variables on the causal pathway. Reperfusion therapies (intravenous thrombolysis and mechanical thrombectomy) were assessed from procedures_icd and prescriptions; these interventions were rarely recorded in the MIMIC-IV tables (thrombolysis 0%, thrombectomy < 0.1%), most likely because acute reperfusion treatments are frequently administered in the emergency department before ICU admission and are incompletely captured in ICU-derived tables [43]. This limitation is addressed in the Discussion.

### Statistical analysis

#### Latent Class Trajectory Model (LCTM) [23–28]

The R package lcmm was used to fit latent class trajectory models with 1–5 classes. Model selection was based on a combination of statistical fit (Bayesian Information Criterion [BIC] and Akaike Information Criterion [AIC]), classification quality (average posterior probability [AvePP] and entropy), minimum class proportion, and clinical interpretability. When statistical fit indices and classification diagnostics were inconsistent, priority was given to classification quality (AvePP) and clinical interpretability. Details of the model selection rationale and fit indices are reported in Results (Sect.  3.2) and Table S1.

#### Missing data handling

Missing data were handled using a prespecified Multiple Imputation by Chained Equations (MICE) approach [44–46], generating 5 imputed datasets. Because the final analytic dataset contained no missing values in the key variables listed in Sect. 2.2, the MICE estimates were identical to a complete-case analysis; both included all 1,562 patients.

#### Survival analysis

Kaplan-Meier survival analysis was used to visualize and compare 28-day survival probabilities across trajectory groups, with the time axis restricted to the 0–28 day follow-up window corresponding to the primary endpoint. Between-group differences were assessed using the log-rank test. Cox proportional hazards regression models [47–49] were used to analyze the association between trajectory groups and 28-day mortality. Four models with stepwise adjustment for confounders were constructed: Model 1 (unadjusted, including only trajectory group); Model 2 (demographics, additionally adjusted for age and sex); Model 3 (disease severity, additionally adjusted for SOFA score, APSIII score, and SAPSII score); and Model 4 (fully adjusted, additionally adjusted for Charlson Comorbidity Index, creatinine, BUN, sodium, potassium, glucose, INR, hemoglobin, white blood cell count, mechanical ventilation, CRRT, and vasopressor use). The Schoenfeld residual test was used to verify the proportional hazards assumption. Adjusted covariates in the fully adjusted model included age, sex, SOFA score, APSIII score, SAPSII score, Charlson Comorbidity Index, creatinine, BUN, sodium, potassium, glucose, INR, hemoglobin, white blood cell count, mechanical ventilation, CRRT, and vasopressor use. Race was not included in the final model (see Sect. 2.3). The proportional hazards assumption was evaluated using Schoenfeld residuals; the global test result is reported in the Fig. 3 legend.

#### Effect decomposition analysis

An exploratory effect decomposition framework [31–36, 50, 51] was employed, with Day 2 platelet as the exposure variable, Delta change (Day 5 − Day 2) as the mediator, and 28-day mortality as the outcome variable. Day 2 platelet was chosen as the exposure (rather than Day 1) to capture the early post-admission platelet level while avoiding immediate admission-day stress-related fluctuations. Bootstrap (*n* = 1,000) was used to estimate confidence intervals.

This baseline-change decomposition has an important structural property: because the mediator is defined as Delta = Day 5 − Day 2, it algebraically contains the negative of the exposure (Day 2 platelet). Consequently, exposure and mediator are structurally correlated (*r* = − 0.255), and the mediator variance is partly a function of the exposure. This shared variance is an inherent feature of baseline-change designs and is methodologically distinct from a mediation model in which the mediator is an independent post-exposure measurement. We therefore do not interpret the decomposition as identifying a causal pathway; rather, it is used as an exploratory tool to quantify how much of the exposure-outcome association can be statistically attributed to concurrent versus subsequent platelet variation.

A second consideration is the interpretation of suppression effects. In this study, the direct effect of Day 2 platelet on mortality was protective (negative on the probability-difference scale), whereas the indirect effect mediated through Delta was harmful (positive). When direct and indirect effects have opposite signs, the total effect can be smaller than either component, and conventional mediation proportion formulas may produce values outside the interpretable 0–100% range. For this reason, we emphasize the direction and statistical significance of the direct and indirect effects rather than the magnitude of the mediation proportion. The analysis should be regarded as exploratory effect decomposition rather than as causal mediation. This effect decomposition is presented as a supplementary exploratory analysis; the primary conclusions are based on the Cox regression and LCTM group comparisons, and these results should not be used to infer specific clinical interventions.

Effect decomposition was implemented using the R mediation package. In the subgroup with Day 2 platelet > 200 × 10⁹/L, patients were classified as Declining (Day 5 − Day 2 < − 20), Stable (− 20 to 20), or Rising (> 20); logistic regression compared mortality odds of Declining versus Stable, adjusting for age, sex, SOFA, and APSIII.

#### Sensitivity analysis

We conducted eight prespecified sensitivity analyses to assess the robustness of the primary findings: (1) complete-case analysis; (2) competing-risk analysis using the Fine-Gray subdistribution hazard model, with discharge alive or transfer as a competing event; (3) ischemic-only analysis, repeating the fully adjusted Cox model after excluding hemorrhagic stroke patients; (4) stroke-subtype-adjusted analysis, adding stroke subtype (ischemic vs. hemorrhagic) as an additional covariate to the fully adjusted Cox model; (5) CRRT-excluded analysis, repeating the fully adjusted Cox model after excluding patients who received continuous renal replacement therapy at any time during the first 5 days after ICU admission; (6) antiplatelet/anticoagulant-adjusted analysis, adding antiplatelet and anticoagulant use to the fully adjusted Cox model; and (7) 5-class LCTM sensitivity analysis, repeating the fully adjusted Cox model using trajectory groups derived from a 5-class LCTM solution; and (8) continuous Day 2 + Day 5 platelet model, entering Day 2 and Day 5 platelet counts as continuous covariates (instead of trajectory group) in the fully adjusted Cox model.

#### Software and significance level

Analyses were performed using R software (version 4.3.1), with main packages including lcmm (version 2.1.0), survival (version 3.5-5), mediation (version 4.5.0), and mice (version 3.16.0). A two-sided *P* < 0.05 was considered statistically significant.

#### Statistical reporting

Continuous variables were tested for normality using the Shapiro-Wilk test or Kolmogorov-Smirnov test. Normally distributed variables were expressed as mean ± standard deviation and compared among groups using one-way ANOVA. Non-normally distributed variables were expressed as median (interquartile range) and compared using the Kruskal-Wallis test. Categorical variables were expressed as n (%) and compared using the χ² test or Fisher’s exact test, as appropriate.

## Results

### Baseline characteristics

A total of 1,562 ICU stroke patients were included (Table 1). The mean age was 67.8 ± 15.1 years, and 52.9% were male. The 28-day mortality was 21.2% (331/1,562).

**Table 1:** Comparison of baseline and clinical characteristics among four trajectory groups

Variable	Overall	High-Declining	Moderate-Low Stable	Low-Rising	Moderate-High Stable	*P* Value	Test
Age, years	67.8 ± 15.1	65.2 ± 14.8	69.7 ± 14.6	57.0 ± 17.1	67.4 ± 14.9	< 0.001	ANOVA
Male, *n* (%)	827 (52.9)	109 (47.6)	468 (57.2)	49 (69.0)	201 (45.3)	< 0.001	Chi-square
Race, *n* (%)						0.653	Chi-square
White	689 (44.1)	102 (44.5)	369 (45.1)	27 (38.0)	191 (43.0)		
non-White	873 (55.9)	127 (55.5)	449 (54.9)	44 (62.0)	253 (57.0)		
ICU Length of Stay, days	9.9 ± 9.0	11.6 ± 9.6	10.1 ± 9.2	9.2 ± 8.8	8.6 ± 8.1	< 0.001	Kruskal-Wallis
Hospital Length of Stay, days	20.2 ± 17.4	21.5 ± 17.6	20.3 ± 18.2	22.1 ± 17.1	19.1 ± 15.9	0.076	Kruskal-Wallis
28-Day Mortality, *n* (%)	331 (21.2)	78 (34.1)	167 (20.4)	9 (12.7)	77 (17.3)	< 0.001	Chi-square
SOFA Score	1.6 ± 2.1	1.6 ± 1.9	1.8 ± 2.3	2.4 ± 2.3	1.1 ± 1.5	< 0.001	ANOVA
APSIII Score	46.4 ± 20.8	52.7 ± 23.7	47.0 ± 21.3	53.2 ± 21.2	40.9 ± 16.5	< 0.001	ANOVA
SAPSII Score	37.9 ± 13.9	41.6 ± 16.4	38.9 ± 13.4	38.4 ± 15.7	34.3 ± 12.1	< 0.001	ANOVA
Charlson Comorbidity Index	1.5 ± 1.3	1.7 ± 1.3	1.6 ± 1.3	1.4 ± 1.3	1.4 ± 1.3	0.005	ANOVA
Baseline Platelet (Day 1), ×10⁹/L	209.8 ± 99.3	316.5 ± 115.6	156.3 ± 55.1	116.1 ± 77.0	268.4 ± 73.0	< 0.001	ANOVA
Day 2 Platelet, ×10⁹/L	191.6 ± 93.4	251.3 ± 123.1	141.3 ± 51.6	142.0 ± 84.0	261.2 ± 71.9	< 0.001	ANOVA
Day 5 Platelet, ×10⁹/L	205.7 ± 96.6	201.2 ± 104.2	151.4 ± 54.0	271.0 ± 140.2	297.8 ± 64.5	< 0.001	ANOVA
Delta (Day 5 - Day 2), ×10⁹/L	14.2 ± 58.1	-50.1 ± 59.6	10.0 ± 32.1	129.0 ± 78.2	36.6 ± 49.0	< 0.001	ANOVA
Creatinine, mg/dL	1.4 ± 1.7	1.7 ± 2.6	1.5 ± 1.5	1.5 ± 2.4	1.2 ± 1.3	< 0.001	Kruskal-Wallis
BUN, mg/dL	25.0 ± 20.5	30.1 ± 25.2	25.7 ± 20.8	26.1 ± 23.1	20.9 ± 15.7	< 0.001	Kruskal-Wallis
Sodium, mmol/L	139.1 ± 4.9	138.4 ± 5.5	139.5 ± 5.0	138.5 ± 5.7	138.6 ± 4.2	< 0.001	ANOVA
Potassium, mmol/L	4.1 ± 0.7	4.3 ± 0.8	4.1 ± 0.7	4.1 ± 0.8	4.1 ± 0.7	0.002	ANOVA
Glucose, mg/dL	155.5 ± 81.8	184.7 ± 125.9	152.2 ± 76.8	160.8 ± 57.4	145.7 ± 58.9	< 0.001	Kruskal-Wallis
INR	1.4 ± 0.6	1.4 ± 0.5	1.4 ± 0.7	1.4 ± 0.3	1.3 ± 0.4	< 0.001	Kruskal-Wallis
Hemoglobin, g/dL	11.2 ± 2.5	11.2 ± 2.5	11.3 ± 2.6	10.1 ± 2.3	11.2 ± 2.3	0.001	ANOVA
White Blood Cell, ×10⁹/L	12.6 ± 10.1	15.2 ± 7.4	11.7 ± 12.4	14.5 ± 10.6	12.5 ± 5.4	< 0.001	Kruskal-Wallis
Heart Rate, bpm	86.8 ± 19.6	90.4 ± 21.0	85.2 ± 19.8	96.6 ± 18.4	86.3 ± 18.0	< 0.001	ANOVA
Temperature, °C	36.8 (36.5–37.1)	36.7 (36.5–37.1)	36.8 (36.5–37.1)	36.9 (36.4–37.7)	36.8 (36.6–37.1)	0.091	Kruskal-Wallis
Mechanical Ventilation, *n* (%)	1311 (83.9)	208 (90.8)	693 (84.7)	68 (95.8)	342 (77.0)	< 0.001	Chi-square
Vasopressors, *n* (%)	599 (38.3)	119 (52.0)	328 (40.1)	34 (47.9)	118 (26.6)	< 0.001	Chi-square
CRRT, *n* (%)	120 (7.7)	31 (13.5)	69 (8.4)	5 (7.0)	15 (3.4)	< 0.001	Chi-square
Stroke Type, *n* (%)						0.541	Chi-square
hemorrhagic	101 (6.5)	10 (4.4)	57 (7.0)	4 (5.6)	30 (6.8)		
ischemic	1461 (93.5)	219 (95.6)	761 (93.0)	67 (94.4)	414 (93.2)		
Antiplatelet Therapy, *n* (%)	1210 (77.5)	181 (79.0)	617 (75.4)	50 (70.4)	362 (81.5)	0.037	Chi-square
Anticoagulant Therapy, *n* (%)	1531 (98.0)	226 (98.7)	802 (98.0)	67 (94.4)	436 (98.2)	0.140	Fisher exact

Data are presented as mean ± standard deviation, median (interquartile range), or frequency (percentage) as appropriate. Delta = Day 5 − Day 2.  *P* values were calculated using one-way ANOVA, Kruskal-Wallis test, or chi-square test as appropriate. Race was coded as 0 = White and 1 = non-White in the source analytic dataset. Stroke subtype, antiplatelet therapy, and anticoagulant therapy were newly extracted from MIMIC-IV v3.1 and added to the revised Table 1. Temperature was non-normally distributed and is reported as median (interquartile range) with a Kruskal-Wallis test

*BUN* Blood Urea Nitrogen, *INR *International Normalized Ratio, *CRRT *Continuous Renal Replacement Therapy

Significant differences in baseline characteristics were observed among trajectory groups. Stroke subtype distribution was similar across the four trajectory groups (*P* = 0.541). Overall, 1,461 patients (93.5%) had ischemic stroke and 101 (6.5%) had hemorrhagic stroke. Antiplatelet therapy was prescribed in 1,210 patients (77.5%) and showed significant heterogeneity across trajectory groups, ranging from 70.4% in the Low-Rising group to 81.5% in the Moderate-High Stable group (*P* = 0.037). Anticoagulant therapy was used in 1,531 patients (98.0%) with no significant difference across groups (*P* = 0.140). Patients in the High-Declining group had higher APSIII scores (52.7 ± 23.7 vs. 47.0 ± 21.3, *P* < 0.001), worse renal function (creatinine 1.7 ± 2.6 vs. 1.5 ± 1.5 mg/dL, *P* < 0.001), and higher rates of mechanical ventilation (90.8% vs. 84.7%, *P* < 0.001) and vasopressor use (52.0% vs. 40.1%, *P* < 0.001) compared with the Moderate-Low Stable group. Notably, SOFA scores differed significantly across the four trajectory groups (*P* < 0.001); for example, the Low-Rising group had a mean SOFA score of 2.4 ± 2.3 compared with 1.1 ± 1.5 in the Moderate-High Stable group.

### Platelet trajectory patterns

**Fig. 1:**
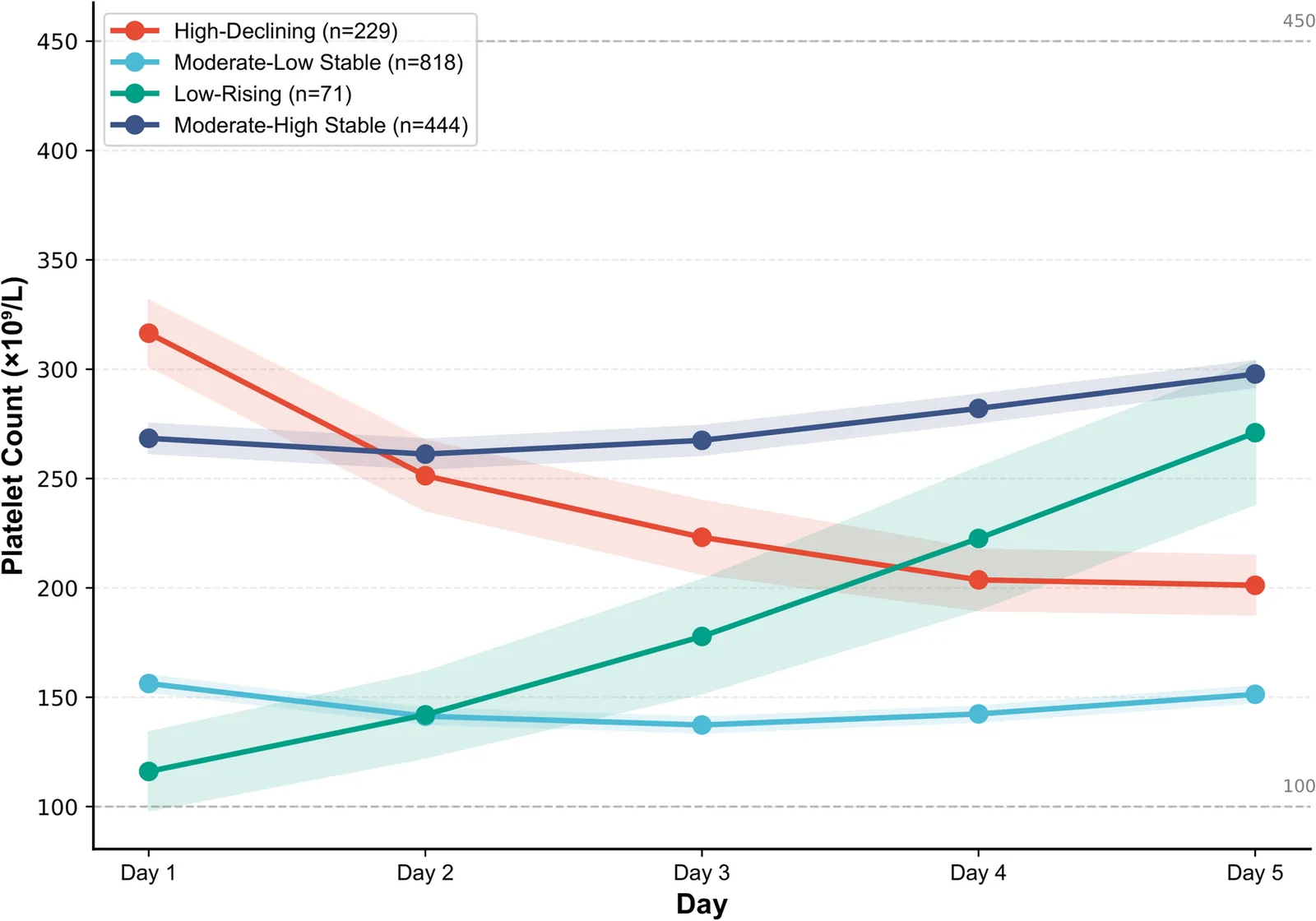
Platelet trajectory patterns identified by Latent Class Trajectory Modeling (LCTM). Four distinct platelet trajectory patterns were identified during the first 5 days of ICU admission in 1,562 stroke patients: (1) High-Declining (*n*=229, 14.7%, red); (2) Moderate-Low Stable (*n*=818, 52.4%, light blue); (3) Low-Rising (*n*=71, 4.5%, green); and (4) Moderate-High Stable (*n*=444, 28.4%, dark blue). Solid lines represent mean platelet counts at each time point; shaded areas represent 95% confidence intervals. Reference lines at 100 and 450 ×10⁹/L indicate thresholds for thrombocytopenia and thrombocytosis, respectively

LCTM identified four distinct platelet trajectory patterns (Fig. 1; complete LCTM fit indices for the 1- to 5-class solutions are provided in Supplementary Table S1):

Model selection considered statistical fit, classification quality, and clinical interpretability. Models with 1–5 classes were fitted; BIC and AIC decreased monotonically as the number of classes increased from 1 to 5. The 4-class model had the highest AvePP (0.91) and a minimum class membership proportion of 14.7% in the LCTM fit. By contrast, the 5-class model showed a lower AvePP (0.82) and the smallest class comprised only 6 patients (0.4%), indicating over-segmentation. Because the 4-class solution provided the best balance of statistical support, high classification quality, and clinically interpretable trajectories, it was selected as the final model. Table S1 presents the AIC, BIC, average posterior probability, entropy, and minimum class proportion for the 1- to 5-class LCTM solutions.

### Mortality comparison

**Fig. 2:**
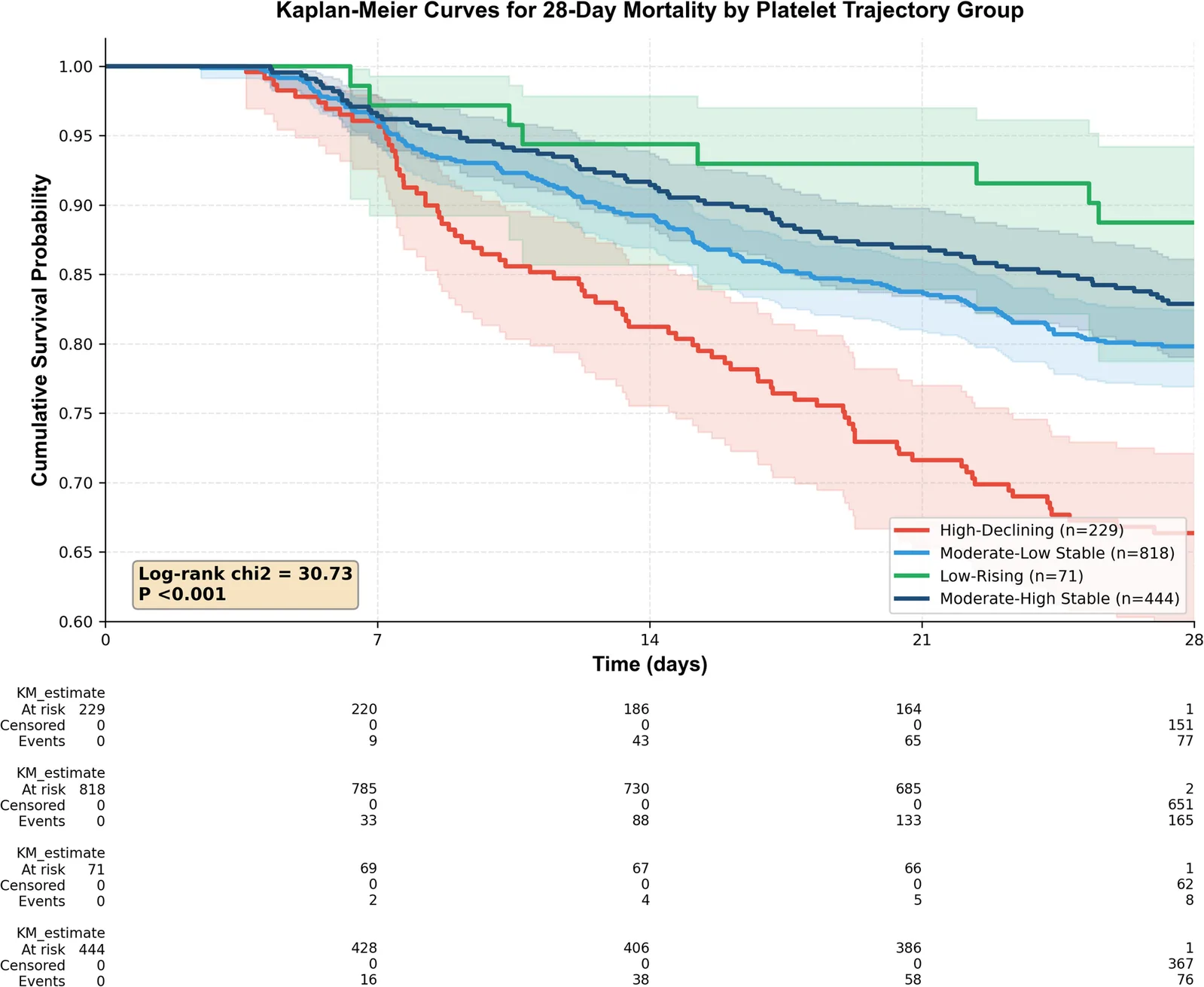
Kaplan-Meier Curves for 28-day mortality by trajectory group. Cumulative survival probabilities are shown for the four platelet trajectory groups over the 0–28 day follow-up period following ICU admission. The High-Declining group (red) demonstrated the lowest survival rate, with a 28-day mortality of 34.1% compared with 20.4% in the Moderate-Low Stable reference group. Between-group comparison was performed using the log-rank test, *P* < 0.001. Shaded areas represent 95% confidence intervals. Number-at-risk values are reported at 0, 7, 14, 21, and 28 days. Survival curves are restricted to the predefined 0–28 day primary follow-up window

Significant differences in 28-day mortality were observed among groups (*P* < 0.001). The High-Declining group had the highest mortality (34.1%), followed by the Moderate-Low Stable group (20.4%), the Moderate-High Stable group (17.3%), and the Low-Rising group (12.7%) (Fig. 2).

### Cox regression analysis

**Fig. 3:**
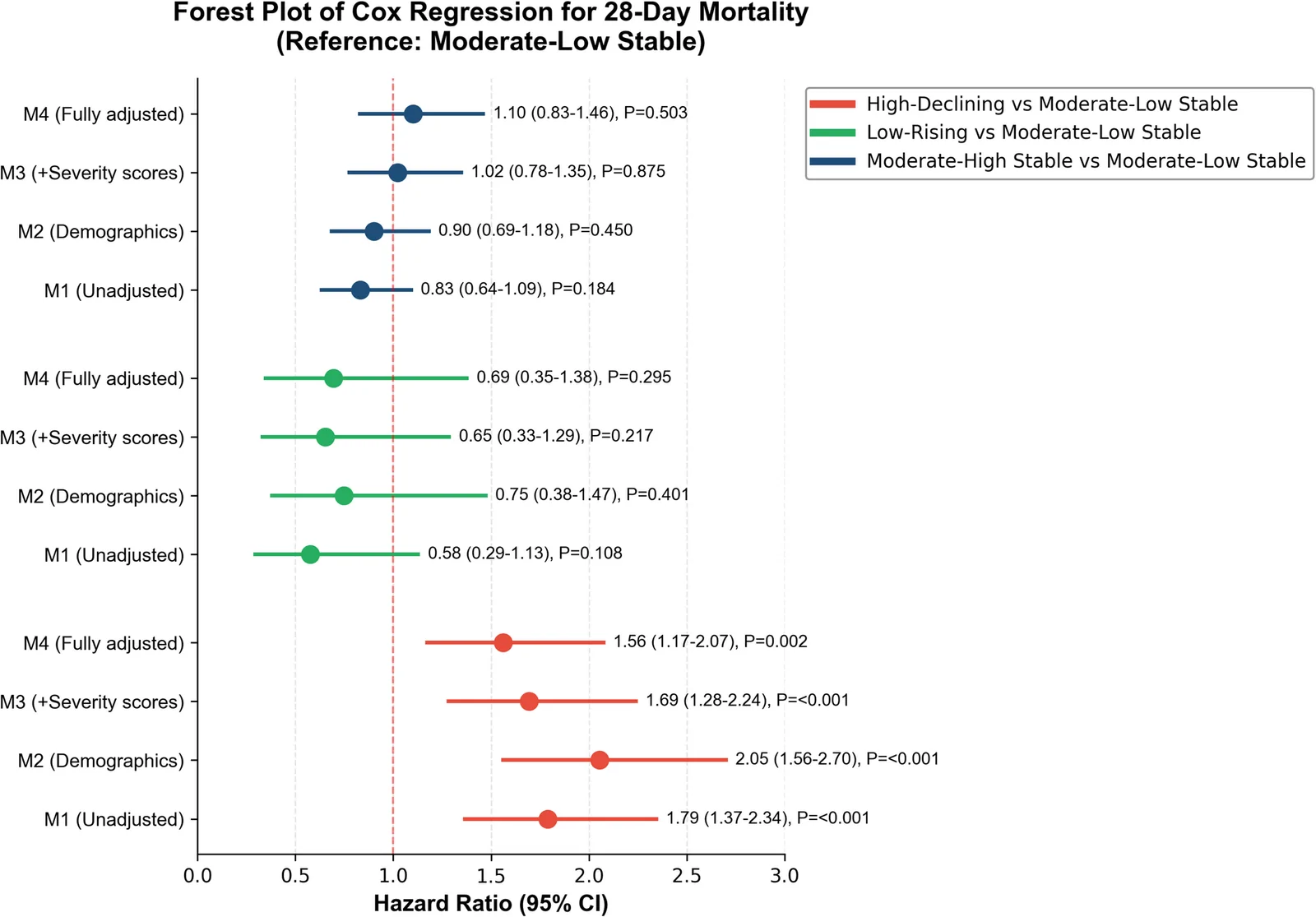
Cox regression forest plot for 28-day mortality. Hazard ratios (HRs) and 95% confidence intervals are presented for three trajectory groups relative to the Moderate-Low Stable group across four models with stepwise adjustment: Model 1 (unadjusted), Model 2 (adjusted for age and sex), Model 3 (additionally adjusted for disease severity scores), and Model 4 (fully adjusted for all covariates). Schoenfeld residual tests showed that all variables satisfied the proportional hazards assumption (global *P* = 0.406). *** *P* < 0.001, ** *P* < 0.01, * *P* < 0.05

The Cox regression results are presented in Table 2; Fig. 3.

**Table 2:** Cox proportional hazards regression analysis results

Model	Comparison	HR (95% CI)	*P* value
Model 1 (Unadjusted)	High-Declining vs. Moderate-Low Stable	1.79 (1.37–2.34)	< 0.001
Model 1 (Unadjusted)	Low-Rising vs. Moderate-Low Stable	0.58 (0.29–1.13)	0.108
Model 1 (Unadjusted)	Moderate-High Stable vs. Moderate-Low Stable	0.83 (0.64–1.09)	0.184
Model 2 (Demographics)	High-Declining vs. Moderate-Low Stable	2.05 (1.56–2.70)	< 0.001
Model 2 (Demographics)	Low-Rising vs. Moderate-Low Stable	0.75 (0.38–1.47)	0.401
Model 2 (Demographics)	Moderate-High Stable vs. Moderate-Low Stable	0.90 (0.69–1.18)	0.450
Model 3 (+ Severity scores)	High-Declining vs. Moderate-Low Stable	1.69 (1.28–2.24)	< 0.001
Model 3 (+ Severity scores)	Low-Rising vs. Moderate-Low Stable	0.65 (0.33–1.29)	0.217
Model 3 (+ Severity scores)	Moderate-High Stable vs. Moderate-Low Stable	1.02 (0.78–1.35)	0.875
Model 4 (Fully adjusted)	High-Declining vs. Moderate-Low Stable	1.56 (1.17–2.07)	0.002
Model 4 (Fully adjusted)	Low-Rising vs. Moderate-Low Stable	0.69 (0.35–1.38)	0.295
Model 4 (Fully adjusted)	Moderate-High Stable vs. Moderate-Low Stable	1.10 (0.83–1.46)	0.503

Model 3 was adjusted for age, sex, SOFA score, APSIII score, and SAPSII score; Model 4 additionally adjusted for Charlson Comorbidity Index, creatinine, BUN, sodium, potassium, glucose, INR, hemoglobin, white blood cell count, mechanical ventilation, vasopressors, and CRRT. ^***P^ < 0.001, ^**P^ < 0.01, ^*P^ < 0.05

The unadjusted model (Model 1) showed that patients in the High-Declining group had a significantly elevated mortality risk (hazard ratio [HR] = 1.79, 95% confidence interval [CI]: 1.37–2.34, *P* < 0.001). After adjusting for age and sex (Model 2), the High-Declining group still showed a significantly increased mortality risk (HR = 2.05, 95% CI: 1.56–2.70, *P* < 0.001). In the model further adjusted for disease severity scores (Model 3), the High-Declining group remained at significantly higher risk (HR = 1.69, 95% CI: 1.28–2.24, *P* < 0.001). In the fully adjusted model incorporating all confounders (Model 4), the High-Declining group still showed a significantly increased mortality risk compared with the Moderate-Low Stable group (HR = 1.56, 95% CI: 1.17–2.07, *P* = 0.002).

### Effect decomposition analysis

Exploratory effect decomposition revealed a suppression pattern: the direct protective effect of higher Day 2 platelet count was offset by an indirect harmful effect mediated through greater subsequent platelet decline (Table 3). The Average Direct Effect (ADE) was − 0.009 (95% CI: −0.033 to 0.015, *P* = 0.452) and the Average Component Mediation Effect (ACME) was 0.017 on the probability-difference scale (95% CI: 0.010–0.026, *P* < 0.001).

**Table 3:** Effect decomposition analysis: impact of baseline platelet and change trend on 28-day mortality

Effect	Estimate (95% CI)	*P* value
ADE (Direct Effect)	-0.009 (-0.033–0.015)	0.452
ACME (Indirect Effect)	0.017 (0.010–0.026)	< 0.001
Total Effect	0.009 (-0.013–0.029)	0.400
Prop. Mediated (%)	1.954	

Based on 1,562 patients. Effect decomposition analysis used the effect decomposition framework on the probability-difference scale; bootstrap *n* = 1,000. Models were adjusted for age, sex, SOFA, and APSIII. Because the direct and indirect effects have opposite directions, the Prop. Mediated estimate of 1.954 is not interpretable as a conventional proportion and is reported for completeness only

*ADE *Average Direct Effect, *ACME *Average Component Mediation Effect

**Fig. 4:**
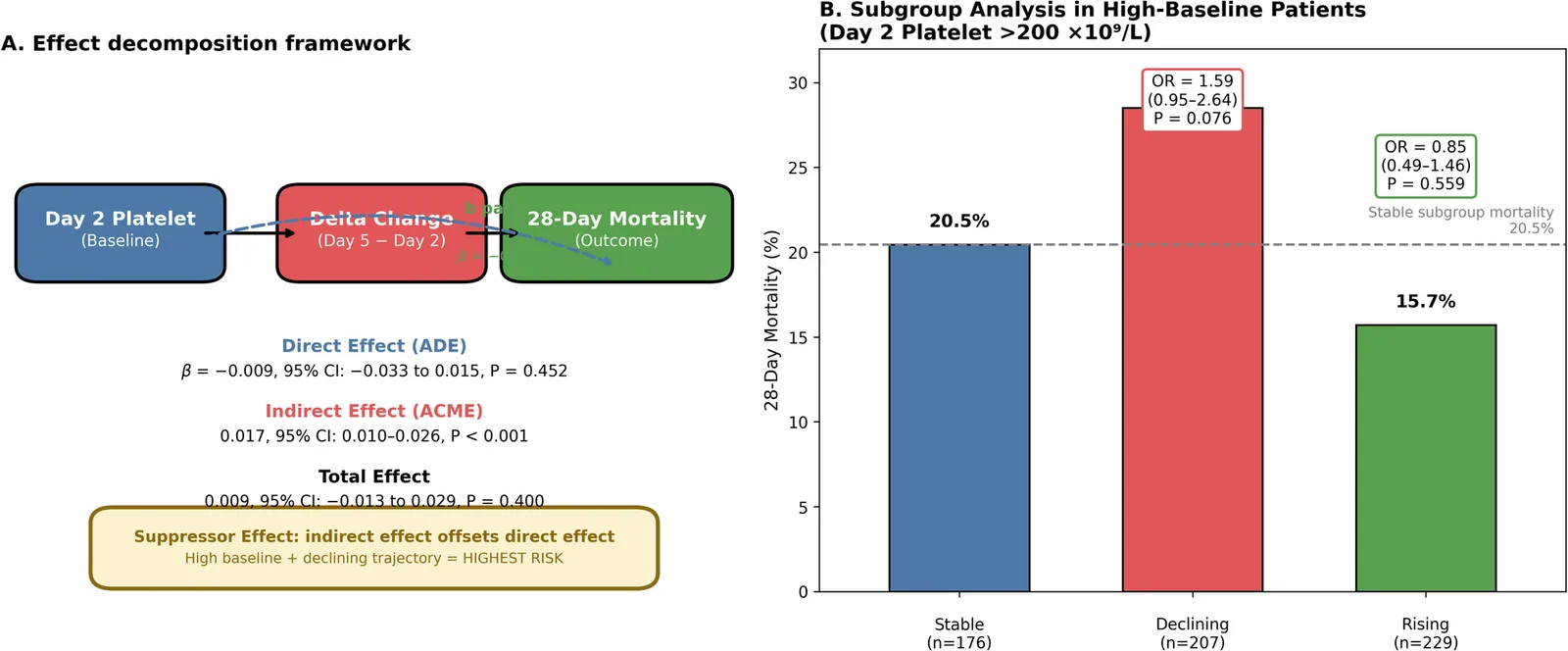
Effect decomposition analysis and subgroup analysis. **A** Effect decomposition framework on the probability-difference scale, showing the decomposition of total effect into direct effect (ADE) and indirect effect (ACME). Day 2 platelet (exposure) → Delta change (Day 5 − Day 2, mediator) → 28-day mortality (outcome). **B** Subgroup analysis in high-baseline patients (Day 2 platelet > 200 × 10⁹/L), showing non-significantly higher mortality odds in the declining trajectory compared with stable trajectories (OR = 1.59, 95% CI: 0.95–2.64, *P* = 0.076). The dashed horizontal line at 20.5% indicates the observed 28-day mortality in the Stable subgroup of high-baseline patients

These effect decomposition results are visualized in Fig. 4A.

Because the direct and indirect effects have opposite directions, conventional mediation proportion formulas yield unreliable absolute mediation proportion values. Consequently, the Prop. Mediated estimate of 1.954 in Table 3 is not interpretable as a conventional proportion and is reported for completeness only; only the direction and statistical significance of the effects are interpreted in this exploratory analysis.

### Sensitivity analysis

In the subgroup with Day 2 platelet > 200 × 10⁹/L, a declining platelet trend (Day 5 − Day 2 < − 20) was associated with a non-significantly higher odds of mortality compared with a stable trend (odds ratio [OR] = 1.59, 95% CI: 0.95–2.64, *P* = 0.076).

The subgroup analysis is presented in Fig. 4B.

To assess the robustness of the primary findings, we conducted eight prespecified sensitivity analyses. In the ischemic-only analysis (*n* = 1,461), the High-Declining group remained associated with a significantly increased 28-day mortality risk (hazard ratio [HR] = 1.62, 95% CI: 1.21–2.18, *P* = 0.001); the full ischemic-stroke subgroup sensitivity results are reported in Supplementary Table S4. Additionally adjusting for stroke subtype (ischemic vs. hemorrhagic) yielded an HR of 1.57 (95% CI: 1.18–2.08, *P* = 0.002), consistent with the primary analysis. After excluding 120 patients who received CRRT, the association remained significant (HR = 1.46, 95% CI: 1.06–2.02, *P* = 0.019). Additional adjustment for antiplatelet and anticoagulant therapy yielded a similar effect estimate (HR = 1.60, 95% CI: 1.20–2.13, *P* = 0.001). In the competing-risk analysis treating discharge alive or transfer as a competing event, the subdistribution hazard ratio [SHR] was 1.65 (95% CI: 1.24–2.18, *P* = 0.0005). Complete-case analysis yielded identical results to the primary analysis because the analytic dataset had no missing key covariates. Finally, when the 5-class LCTM solution was used, the High-Declining-like class (class 4, *n* = 6, 0.4%) showed a point estimate in the same direction as the primary analysis but with wide confidence intervals (HR = 1.41, 95% CI: 0.35–5.77, *P* = 0.63), reflecting over-segmentation and instability of the smallest class. In a continuous Day 2 + Day 5 platelet model, higher Day 2 platelet (HR = 1.004 per 1 × 10⁹/L, 95% CI: 1.002–1.006, *P* < 0.001) and lower Day 5 platelet (HR = 0.996 per 1 × 10⁹/L, 95% CI: 0.994–0.998, *P* < 0.001) were independently associated with higher mortality, consistent with the high-baseline/declining phenotype. Across all analyses, the effect size, direction, and statistical significance of the High-Declining association were consistent with the primary analysis (Model 4 HR = 1.56, 95% CI: 1.17–2.07, *P* = 0.002), indicating that the main conclusion was robust; all prespecified sensitivity analyses are summarized in Supplementary Table S5.

## Discussion

Only limited prior research has explored sequential platelet trajectory patterns among ICU stroke patients.

This study has the following strengths: (1) we applied LCTM to dynamic platelet analysis in ICU stroke patients, revealing four distinct platelet change patterns from the perspective of population heterogeneity; (2) it combines effect decomposition analysis, quantifying how the indirect effect of baseline platelet levels may operate through subsequent platelet decline, providing exploratory evidence for characterizing the association between platelets and mortality.

### Clinical significance of platelet trajectory patterns

Even among patients with normal or near-normal initial platelet counts, the direction and rate of early change were associated with 28-day mortality. This suggests that the platelet trend captures evolving pathophysiology that a static measurement may miss.

The four trajectory groups illustrate distinct prognostic phenotypes. The High-Declining group, despite an initial platelet count at the upper-normal range, experienced the largest relative decline and the highest mortality, underscoring that early loss of platelets may signal consumption, bone-marrow suppression, or treatment-related effects in critically ill stroke patients. By contrast, the Low-Rising group began with low counts but recovered substantially and had the lowest mortality. This pattern is hypothesis-generating and could be consistent with resolution of an acute stress response or effective supportive care, but other explanations cannot be excluded. This trajectory should be interpreted as a prognostic stratification signal only and does not support reduced monitoring intensity or altered clinical vigilance without prospective validation. The two stable trajectories occupied intermediate positions, with the Moderate-High Stable group showing marginally lower mortality than the Moderate-Low Stable group. These patterns should be interpreted as prognostic stratification signals rather than as specific pathophysiological diagnoses or direct therapeutic targets.

Several mechanisms could underlie a declining platelet trajectory in this setting, including disseminated intravascular coagulation, microthrombosis, hemodilution from fluid resuscitation or transfusion, bone-marrow suppression secondary to severe infection or systemic inflammation, heparin-induced thrombocytopenia, and bleeding-related consumption. Our observational design does not allow us to distinguish among these possibilities. Consequently, a rapid platelet decline should prompt clinical evaluation for reversible causes such as bleeding, sepsis, disseminated intravascular coagulation, or heparin-induced thrombocytopenia, but it does not establish a causal pathway or a specific indication for treatment based on this study alone.

### Implications of effect decomposition analysis

Effect decomposition analysis revealed that higher baseline platelet levels showed a protective direct effect on 28-day mortality, although this direct effect did not reach statistical significance (ADE = − 0.009, *P* = 0.452). This exploratory analysis found that among high-baseline patients, a declining platelet trend was associated with a non-significantly higher odds of mortality (odds ratio [OR] = 1.59, 95% CI: 0.95–2.64, *P* = 0.076). This pattern is consistent with the highest mortality rate observed in the High-Declining trajectory group. Because the mediator structurally contains the exposure, these estimates should not be interpreted as evidence of a causal pathway; they only describe how the exposure-outcome association is statistically partitioned.

### Potential clinical implications

These are hypothesis-generating suggestions for future prospective validation, not practice-changing recommendations.

Based on the findings of this study, the following potential clinical implications are outlined: (1) platelet trajectory patterns may serve as prognostic markers, but they are not established therapeutic targets; (2) for patients with platelet counts > 200 × 10⁹/L at admission, a declining trend identifies a high-risk phenotype and may prompt clinicians to evaluate for potential underlying causes such as bleeding, sepsis, DIC, or HIT; and (3) patients with platelet counts < 100 × 10⁹/L but a rising trend had the lowest mortality in this cohort, although this observation requires validation before influencing clinical decisions.

### Sensitivity analysis

To verify the robustness of the main findings, sensitivity analyses were conducted (Fig. S1, Supplementary Table S5). Because the current 28-day analysis dataset contained no missing values in key variables, complete-case analysis included all 1,562 patients and yielded identical results to MICE multiple imputation, indicating that missing data had no impact on the results. The trajectory group distribution was: High-Declining 229 (14.7%), Moderate-Low Stable 818 (52.4%), Low-Rising 71 (4.5%), and Moderate-High Stable 444 (28.4%) in both analyses, further validating the stability of the trajectory model. After excluding the 101 hemorrhagic stroke patients, the fully adjusted Cox model yielded an HR of 1.62 (95% CI: 1.21–2.18, *P* = 0.001) for High-Declining vs. Moderate-Low Stable, confirming that the main findings were not driven by the inclusion of hemorrhagic stroke patients. Exclusion of 120 patients who received CRRT yielded an HR of 1.46 (95% CI: 1.06–2.02, *P* = 0.019). Additional adjustment for antiplatelet and anticoagulant therapy yielded an HR of 1.60 (95% CI: 1.20–2.13, *P* = 0.001), despite the near-universal use of anticoagulants (98.0%). A competing-risk analysis treating discharge alive or transfer as a competing event yielded consistent results (SHR = 1.65, 95% CI: 1.24–2.18, *P* = 0.0005; Supplementary Table S3). Finally, a 5-class LCTM sensitivity analysis produced a High-Declining-like class of only 6 patients (0.4%) with an unstable HR estimate, supporting the decision to use the more parsimonious 4-class model. A continuous Day 2 + Day 5 platelet model showed that higher Day 2 platelet (HR = 1.004 per 1 × 10⁹/L, 95% CI: 1.002–1.006, *P* < 0.001) and lower Day 5 platelet (HR = 0.996 per 1 × 10⁹/L, 95% CI: 0.994–0.998, *P* < 0.001) were independently associated with higher mortality, consistent with the high-baseline/declining phenotype.

### Study limitations

This study has several inherent limitations common to retrospective observational research. First, the causal relationship between platelet trajectories and mortality should be interpreted with caution because reverse causality may exist: clinical deterioration can lead to platelet decline rather than platelet decline causing death. Second, despite adjustment for multiple confounders, residual confounding from unmeasured or imprecisely measured factors cannot be excluded. Third, the effect decomposition analysis assumes no unmeasured confounding of the exposure-mediator, mediator-outcome, and exposure-outcome relationships; this assumption is unlikely to be fully satisfied in routinely collected ICU data.

MIMIC-IV is a single-center US critical care database; therefore, the findings may not be directly generalized to healthcare systems outside the United States or to clinical settings with different patient demographics and practice patterns. Furthermore, because the cohort comprised 93.5% ischemic stroke and only 6.5% hemorrhagic stroke patients, the findings should be interpreted as primarily reflecting ischemic stroke and should not be generalized to hemorrhagic stroke or subarachnoid hemorrhage. In addition, stroke was the primary admission diagnosis in only 48.6% of patients, with the remaining 51.4% recorded as a secondary diagnosis, reflecting MIMIC-IV’s ICU-centric structure. These case-ascertainment patterns should be considered when generalizing the findings.

Several methodological limitations affect causal interpretation. First, ICU interventions (mechanical ventilation, vasopressors, CRRT, antiplatelet/anticoagulant therapy) and underlying disease severity evolve dynamically over the first days after admission. Patients with deteriorating platelet trajectories may receive more intensive treatments, and standard Cox models cannot fully capture this time-varying confounding; residual confounding by indication therefore remains possible. Second, LCTM assigns each patient to the most likely trajectory class based on posterior probabilities. Although the average posterior probability in our final model was high (0.91), it was not 1; consequently, individual-level class membership carries probabilistic uncertainty and should be interpreted with caution. Third, the identified platelet trajectories should be regarded as prognostic biomarkers or markers of disease severity rather than as independent causal mediators of mortality.

We could not perform dedicated sensitivity analyses for massive blood transfusion, advanced liver disease, or hypersplenism because standardized recording of transfusion volume, administration timing, and hepatic disease severity is absent in the MIMIC-IV ICU tables used for extraction. These factors independently alter platelet production and consumption, introducing unmeasured residual confounding. Furthermore, platelet function indices (such as PFA-100 and thromboelastography) were not included, precluding assessment of changes in platelet activity. Finally, this study could not reliably capture acute reperfusion therapies (intravenous thrombolysis and mechanical thrombectomy) because MIMIC-IV prescriptions and procedures tables primarily record ICU-era data; these interventions are frequently administered in the emergency department before ICU admission and were rarely documented in our extraction (thrombolysis 0%, thrombectomy < 0.1%), consistent with reports from other MIMIC-IV stroke studies [43].

### Conclusions

Dynamic platelet trajectory patterns are associated with mortality in ICU stroke patients and may help identify high-risk phenotypes. The High-Declining group showed a significantly increased mortality risk. Exploratory effect decomposition analysis indicated that for patients with high baseline platelet levels, subsequent platelet decline contributes to the highest-risk phenotype. These findings should be interpreted as identifying a prognostic pattern rather than a causal pathway; any clinical application requires confirmation in future prospective studies.

## Supplementary Information

Additional file 1: Table S1. Latent Class Trajectory Model Fit Indices. Comparison of model fit indices for latent class trajectory models with 1–5 classes, including AIC, BIC, log-likelihood, average posterior probability (AvePP), minimum class proportion, and model selection.

Additional file 2: Table S2. Comparison of Baseline Characteristics Between Ischemic and Hemorrhagic Stroke Patients. Baseline demographics, disease severity, laboratory parameters, treatments, and 28-day mortality in ischemic (n=1,461) versus hemorrhagic (n=101) stroke patients.

Additional file 3: Table S3. Fine-Gray Subdistribution Hazard Ratios for 28-Day Mortality. Competing-risk analysis treating discharge alive or transfer as a competing event.

Additional file 4: Table 4. Cox Sensitivity Analysis After Excluding Hemorrhagic Stroke Patients. Fully adjusted Cox model results in the ischemic-only cohort (*n*=1,461).

Additional file 5: Table S5. Summary of Sensitivity Analyses. Summary of all prespecified sensitivity analyses, including primary analysis, ischemic-only analysis, CRRT exclusion, additional adjustment for antiplatelet and anticoagulant therapy, additional adjustment for stroke subtype, competing-risk analysis, complete-case analysis, 5-class LCTM solution, and continuous Day 2 + Day 5 platelet model.

Additional file 6: Figure S1. Sensitivity Analysis: MICE Imputation vs Complete-Case Analysis. Comparison of trajectory group distribution, baseline characteristics, 28-day mortality, and ICU length of stay between MICE multiple imputation and complete-case analyses.

Additional file 7: Figure S2. Patient Selection Flowchart (CONSORT Style). Flow diagram showing the patient selection process from the MIMIC-IV database (version 3.1) to the final analytic cohort of 1,562 ICU stroke patients.

## Data Availability

The MIMIC-IV database (version 3.1) used in this study is publicly available via PhysioNet (https://physionet.org/content/mimiciv/3.1/). Access to the database requires completion of the PhysioNet Credentialing Program and signing of the Data Use Agreement. The data supporting the findings of this study are available from the corresponding author upon reasonable request.

## Abbreviations

ACME: Average Component Mediation Effect
ADE: Average Direct Effect
AIC: Akaike Information Criterion
APSIII: Acute Physiology Score III
AvePP: Average posterior probability
BIC: Bayesian Information Criterion
BIDMC: Beth Israel Deaconess Medical Center
BUN: Blood urea nitrogen
CI: Confidence interval
CRRT: Continuous renal replacement therapy
HIPAA: Health Insurance Portability and Accountability Act
HR: Hazard ratio
ICU: Intensive care unit
ICD: International Classification of Diseases
INR: International normalized ratio
IRB: Institutional Review Board
LCTM: Latent class trajectory models
MICE: Multiple Imputation by Chained Equations
MIMIC-IV: Medical Information Mart for Intensive Care iv
OR: Odds ratio
SAPSII: Simplified Acute Physiology Score II
SHR: Subdistribution hazard ratio
SOFA: Sequential Organ Failure Assessment
